# Utilizing of Adsorptive Transfer Stripping Technique Brdicka Reaction for Determination of Metallothioneins Level in Melanoma Cells, Blood Serum and Tissues

**DOI:** 10.3390/s8053106

**Published:** 2008-05-10

**Authors:** Sona Krizkova, Ivo Fabrik, Vojtech Adam, Jiri Kukacka, Richard Prusa, Grace J. Chavis, Libuse Trnkova, Jan Strnadel, Vratislav Horak, Rene Kizek

**Affiliations:** 1 Department of Chemistry and Biochemistry, Zemedelska 1, CZ-613 00 Brno, Czech Republic; 2 Department of Animal Nutrition and Forage Production, Faculty of Agronomy, Mendel University of Agriculture and Forestry, Zemedelska 1, CZ-613 00 Brno, Czech Republic; 3 Department of Clinical Biochemistry and Pathobiochemistry, 2nd Faculty of Medicine, Charles University, V Uvalu 84, CZ-150 06 Prague 5, Czech Republic; 4 Department of Chemistry, University of California, One Shields Avenue, CA-956 16 Davis, USA; 5 Department of Chemistry, Faculty of Science, Masaryk University, Kotlarska 2, CZ-611 37 Brno, Czech Republic; 6 Laboratory of Tumour Biology, Department of Animal Embryology, Cell and Tissue Differentitation, Institute of Animal Physiology and Genetics, Academy of Sciences of the Czech Republic, v.v.i., CZ-277 21 Libechov, Czech Republic

**Keywords:** Metallothionein, Protein, Tumour Marker, Cell, Animal Tissue, Human blood serum, Differential Pulse Voltammetry, Adsorptive Transfer Stripping Technique, Brdicka Reaction

## Abstract

In the paper we utilized the adsorptive transfer stripping differential pulse voltammetry Brdicka reaction for the determination of metallothioneins (MT) in melanoma cells, animal melanoma tissues (MeLiM miniature pig) and blood serum of patients with malignant melanoma. Primarily we attempted to investigate the influence of dilution of real sample on MT electrochemical response. Dilution of samples of 1 000 times was chosen the most suitable for determination of MT level in biological samples. Then we quantified the MT level in the melanoma cells, the animal melanoma tissues and the blood serum samples. The MT content in the cells varied within the range from 4.2 to 11.2 μM. At animal melanoma tissues (melanomas localized on abdomen, back limb and dorsum) the highest content of MT was determined in the tumour sampled on the back of the animal and was nearly 500 μg of MTs per gram of a tissue. We also quantified content of MT in metastases, which was found in liver, spleen and lymph nodes. Moreover the average MT level in the blood serum samples from patients with melanoma was 3.0 ± 0.8 μM. MT levels determined at melanoma samples were significantly (p < 0.05) higher compared to control ones at cells, tissues and blood serum.

## 1. Introduction

Metallothioneins (MT) are a group of low molecular mass (about 6.5 kDa) single-chain proteins rich in cysteine and free of aromatic aminoacids [1-3]. MT are a family of proteins with a large degree of sequence homology, which have been described in bacteria, fungi, plants, and animal species [1,4-7]. Four major isoforms (MT-1 through MT-4) have been identified in mammals. In addition at least thirteen known closely related MT proteins in humans have been described. However the specific functional roles of MTs isoforms and their molecular interactions are still unclear [1]. In the last decade, several reports disclosed MT expression as a useful diagnostic factor for tumour progression and drug resistance in a variety of malignancies e.g. leukaemia, melanoma, breast, ovarian, renal, lung, pancreatic, gall bladder, oesophageal, and basal cell carcinomas [8-27].

The number of patients with malignant melanoma has increased markedly during last decades; therefore new markers of the tumour disease need to be search to improve its diagnostic and therapy. Several articles reported on the monitoring the association between MT level and course of the disease at patients with malignant melanoma have been published [19,25,27-30]. The last study on 1 270 patients showed the faster progression of the disease at patients with the higher MT level [28]. The exact biological role of MT in melanoma is still unclear. One the current accepted opinion is that MT is involved in intergrowing of melanoma to surrounding tissues and in scavenging of reactive oxygen species in melanocytes [31]. The malignant melanoma is etiologically heterogeneous disease [32]. The inherited melanoma was firstly diagnosed in 1820. It was shown that the better survival was observed at inherited type of melanoma compared to non-inherited. Four genes respondent for four types of inherited melanoma have been identified - cutaneous malignant melanoma 1 (CMM1) on chromosome 1p36, cutaneous malignant melanoma 2 (CMM2) gene CDKN2A on chromosome 9p21, cutaneous malignant melanoma 3 (CMM3) gene CDK4 on 12q14 and cutaneous malignant melanoma 4 CMM4 on chromosome 1p22 [33-37]. Melanoma can be also diagnosed at many syndromes affiliated with chromosomal instability like *anaxia telangiectasi*, Nijmengen breakage syndrome, Fanconi anaemia, Bloom syndrome or *xeroderma pigmentosum*.

The methods used for determination of MT in clinical samples are often based on relatively time-consuming and not enough sensitive immunohistochemistry [27,38,39]. There can be used also spectrometric [40-42], chromatographic [43-45] or electrochemical techniques for determination of MT [46-49]. From the wide spectrum of electrochemical methods used for determination of MT differential pulse voltammetry [49-64] and/or chronopotentiometric stripping analysis [46,65-77] are the most sensitive ones. To investigate the role of MT in melanoma development we employed the adsorptive transfer stripping differential pulse voltammetry Brdicka reaction for the determination of these proteins in melanoma cells, animal melanoma tissues (MeLiM miniature pig) and blood serum of patients with malignant melanoma.

## 2. Material and Methods

### 2.1 Chemicals and pH measurements

Rabbit liver MT (MW 7143 g·mol^-1^), containing 5.9 % Cd and 0.5 % Zn, was purchased from Sigma Aldrich (St. Louis, USA). Co(NH_3_)_6_Cl_3_ and other chemicals used were purchased from Sigma Aldrich (Sigma-Aldrich, USA) unless noted otherwise. The stock standard solutions of MT (10 μg·ml^-1^) was prepared with ACS water (Sigma-Aldrich, USA) and stored in the dark at −20 °C. Working standard solutions were prepared daily by dilution of the stock solutions with ACS water. The primary mouse anti-metallothionein antibody and the rabbit anti-mouse immunoglobulin-HRP conjugate were from Dako (Dako, Denmark). The pH value was measured using WTW inoLab Level 3 with terminal Level 3 (MultiLab Pilot; Weilheim, Germany).

### 2.2 Melanoma Cell lines

Primary melanoma cell lines wM4, wM9 and metastatic melanoma cell lines wM12 and wM15 were obtained from Masaryk Memorial Cancer Institute in Brno, Czech Republic. These lines are considered to be stable in *in vitro* conditions, other details in ref. No. [78].

### 2.3 Animals

The miniature pigs are housed in the Institute of Animal Physiology and Genetics of the Academy of Sciences of the Czech Republic, Libechov. The experimental herd of laboratory pigs was founded by importation of 5 animals of the Hormel strain from the USA in 1967. These animals were crossbred for porcine blood group studies with several other breeds or strains: Landrace, Large White, Cornwall, Vietnamese pigs and miniature pigs of the Gottingen origin. Different cross-breeding produced more than 2000 descendants without any signs of melanoma. Nevertheless, a few black piglets with melanoma had occurred in this genetically heterogeneous population by 1989. They originated from mating two male brothers with four related sows. These parents had no visible skin tumours. The MeLiM strain with hereditary melanoma was established using selective breeding. The skin nodular melanomas used in this study were taken from abdomen (E3-1/1), hind limb (E3-1/3) and back (E3-1/4) of one highly affected animal with progressing tumours shortly before death (in age of 1 month). Moreover, the metastases from cervical lymph node (E3-1/5), spleen (E3-1/6), lungs (E3-1/7) and liver (E3-1/8) were analysed.

### 2.4 Patients with malignant melanoma

Blood serum samples were obtained from the Department of Clinical Biochemistry and Pathobiochemistry, 2^nd^ Faculty of Medicine Charles University, Czech Republic. The sample was prepared by heat treatment and solvent precipitation.

### 2.5 Preparation of biological samples for electrochemical analysis

The harvested cells or animal tissue (app. 0.2 g) were transferred to a test tube and then deep frozen by liquid nitrogen to disrupt cells. The frozen cells were mixed with extraction buffer (100 mM potassium phosphate, pH 8.7) to a final volume of 1 ml and homogenised using hand-operated homogenizer ULTRA-TURRAX T8 (IKA, Germany) placed in an ice bath for 3 min at 25,000 rpm [79]. The homogenate was centrifuged at 10 000 g for 15 min and at 4°C (Eppendorf 5402, USA).

The processed cells or tissues, or blood serum samples were prepared by heat treatment. Briefly, the sample was kept at 99 °C in a thermomixer (Eppendorf 5430, USA) for 15 min. with occasional stirring, and then cooled to 4 °C. The denatured homogenates were centrifuged at 4 °C, 15 000 *g* for 30 min. (Eppendorf 5402, USA). Heat treatment effectively denatures and removes high molecular weight proteins out from samples [80].

### 2.6 Apparatus

#### 2.6.1 Electrochemical measurements

Electrochemical measurements were performed with AUTOLAB Analyzer (EcoChemie, Netherlands) connected to VA-Stand 663 (Metrohm, Switzerland), using a standard cell with three electrodes. A hanging mercury drop electrode (HMDE) with a drop area of 0.4 mm^2^ was employed as the working electrode. An Ag/AgCl/3M KCl electrode served as the reference electrode. Glassy carbon electrode was used as the auxiliary electrode. For smoothing and baseline correction the software GPES 4.9 supplied by EcoChemie was employed.

#### 2.6.2 Adsorptive transfer stripping technique differential pulse voltammetry Brdicka reaction of MT

Principle of the adsorptive transfer stripping technique (AdTS) is based on the strong adsorbing of the target molecule on the electrode surface at an open electrode circuit [81]. The electrode is washed in a rinsing buffer. The electrode is further transferred to the supporting electrolyte and measured. The Brdicka supporting electrolyte containing 1 mM Co(NH_3_)_6_Cl_3_ and 1 M ammonia buffer (NH_3_(aq) + NH_4_Cl, pH = 9.6) was used; surface-active agent was not added. The samples of the MT were reduced before each measurement by 1 mM tris(2-carboxyethyl)phosphine addition according to [46,56]. AdTS DPV Brdicka reaction parameters were as follows: an initial potential of −0.35 V, an end potential −1.8 V, a modulation time 0.057 s, a time interval 0.2 s, a step potential of 1.05 mV, a modulation amplitude of 250 mV, E_ads_ = 0 V. AdTS DPV parameters were as follows: an initial potential of −1.2 V, an end potential −0.3 V, a modulation time 0.057 s, a time interval 0.2 s, a step potential of 1.05 mV, a modulation amplitude of 250 mV, E_ads_ = 0 V. All experiments were carried out at 4 °C (Julabo F12, Germany).

#### 2.6.3 Dot Immunobinding Assay (DIA)

For immunobinding assay the PVDF membrane (Bio-Rad) was used. The sample (1 μl) was applied and dried. Further the membrane was blocked in 2% BSA in PBS (137 mM NaCl, 2.7 mM KCl, 1.4 mM NaH_2_PO_4_, 4.3 mM Na_2_HPO_4_, pH 7.4) for 0.5 h with constant shaking. The incubation with primary antibody in dilution of 1:200 was carried out for 1h at 37 °C. After the three times repeated washing in 0.05% PBS-T for 5 min the membrane was incubated in the presence of secondary antibody in dilution 1:1500 for 1h at 37 °C. Then the membrane was washed three times in 0.05% PBS-T for 5 min and incubated in chromogenic substrate (0.4 mg·ml^-1^ AEC (3-aminoethyl-9-carbazole) in 0.5 M acetate buffer with 0.1 % H_2_O_2_, pH 5.5). After the sufficient colouring the reaction was stopped by rinsing in water. The dot intensity was evaluated densitometricaly by Biolight software (Vilber-Lourmat).

#### 2.6.4 Cell counting

Counting of BY-2 suspension cells was carried out using a Fuchs-Rosenthal haemocytometer (Germany). Aliquots of suspension were diluted with distilled water and loaded into the heamocytometer according to the instructions of the manufacturer. The counting of cells was performed manually using a microscope (Olympus, Japan).

### 2.7 Statistical analyses

Data were processed using MICROSOFT EXCEL® (USA). Results are expressed as mean ± S.D. unless noted otherwise. Statistical significance of the differences between MT level quantified in control and melanoma samples was determined. Differences with p < 0.05 were considered significant (t-test was applied for means comparison).

## 3. Results and Discussion

### 3.1 Influence of dilution and kind of real sample on MT signal

**Figure 1.**
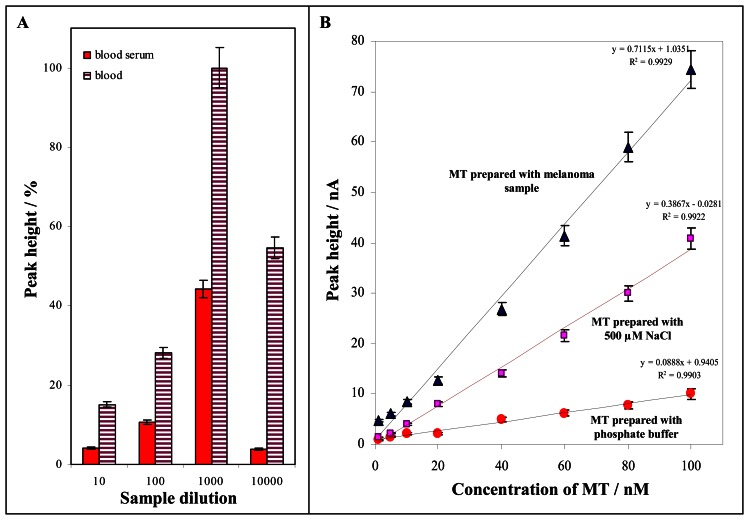
Influence of the dilution of blood serum and blood on the Cat2 peak height (**A**). Dependence of Cat2 peak height on concentration of MT prepared with 1 000 times diluted blood serum sample, 500 μM NaCl and 0.2 M, pH 6.8 phosphate buffer.

Based on the abovementioned results we attempted to investigate the influence of a real sample naturally containing surface active agents and NaCl on Cat2 peak height. The investigation was carried out measuring by differently diluted samples of blood serum or blood of healthy volunteer prepared according to procedure described in “Material and Methods” section. The samples were diluted by 0.2 M phosphate buffer (pH = 6.8). It clearly follows from the results obtained that the Cat2 peak height enhanced with dilution of the sample up to 1 000 fold dilution. The signal decreased in the more dilution sample for both blood serum and blood due to lower content of MT determined (Fig. 3A). Therefore we aimed our attention on the analysis of less diluted samples (10 ×, 100 ×, 250 ×, 500 × and 1 000 × diluted), where the composition of samples markedly influenced the measurements. The effect of the less diluted samples on MT determination was investigated by the method of MT standard addition (100 nM MT was added to the samples). The lowest Cat2 signal was determined at 10 and 100 times diluted samples, where, the recoveries were 15 and 40 %, respectively. The recoveries estimated in the samples diluted 250 and 500 times were 55 and 75 %, respectively. The recovery determined in the 1 000 times diluted samples was 90 %. The best recovery (95 %) was estimated in the 10 000 times diluted samples, but the samples with lower content of MT could not been analysed, if we used such lower dilution ratio. Considering this fact and the very good ratio between recovery and signal height 1 000 times dilution of samples was chosen the most suitable for determination of MT level in biological samples (Fig. 1A).

In the following experiments the Cat2 peak height dependences on MT concentration (1, 5, 10, 20, 40, 60, 80 and 100 nM) were measured, whereas the MT working solutions were prepared with various types of solutions (1 000 times diluted melanoma sample, phosphate buffer and NaCl 500 μM). The calibration curve of MT prepared with phosphate buffer (0.2 M, pH 6.8) was linear with equation of y = 0.0888x + 0.9405, R^2^ = 0.9903 (Fig. 1B). If we investigated the changes of the Cat2 peak height of MT prepared with melanoma sample 1000 times diluted with phosphate buffer, the obtained calibration curve was linear (y = 0.7115x + 1.0351 R^2^ = 0.9929). When the matrix of biological sample was present, the slope of the studied concentration dependence was more than 8 times higher compared with the dependence measured with the sample prepared with the phosphate buffer (Fig. 1B). This begs the question whether the observed Cat2 signal increase can be associated with the higher concentration of ions e.g. NaCl (higher ionic strength). Therefore the different concentrations of NaCl were added (100, 500 and 1 000 μM) to the MT standard (100 nM) prepared with the phosphate buffer. All NaCl additions resulted in the increase of Cat2 peak height. NaCl of 500 μM concentration was chosen to prepare the calibration curve. The dependence obtained was linear y = 0.3867x - 0.0281, R^2^ = 0.9922. The slope of the dependence is four times higher compared to the dependence of MT prepared with phosphate buffer (Fig. 1B). Based on the results obtained the higher ionic strength due to presence of NaCl well mimic the affecting of Cat2 peak height by real sample composition.

### 3.2 Detection of MT in tumour cell cultures

**Figure 2.**
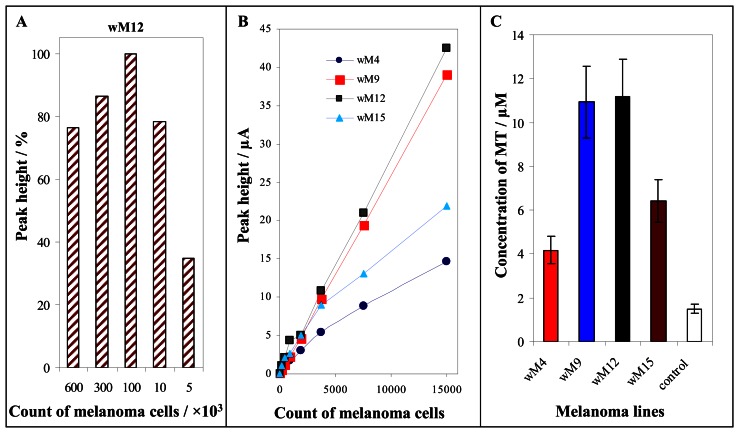
The change of Cat2 peak height with increasing count of cells of cell line wM12 (5, 10, 100, 300 and 600 × 10^3^). The different cell counts were diluted and homogenized in resulting volume of 100 μl (**A**). The dependence of Cat2 peak height on cell counts from 0 to 15 thousands cells (**B**). MT content at single cell lines. Ten thousands of the cells was homogenized in 100 μl of phosphate buffer and then analyzed. The concentration was derived from the calibration curve (**C**).

The optimized electroanalytical method was further utilized for MT determination in various types of biological samples. Primarily we determined MT in tumour cell lines the primary cell culture of malignant melanoma and melanoma cell culture derived from metastases. The samples of four cell lines (wM4, wM9, wM12 and wM15) were diluted on cell counts of 5 000, 10 000, 100 000 and 600 000 cells in 100 μl and prepared according to procedure mentioned in “Materials and Methods” section. The dependence of Cat2 peak height on cell number for line wM12 is shown in Fig. 2A. The dependence obtained has a maximum at 100 000 cells in the sample. At higher cell count the MT signal was about 15 % lower compared to Cat2 signal obtained for 100 000 cells (Fig. 2A). This phenomenon can relate with non-linear response of the method on high content of MT. If the cell number was lower than 100 000, the linear decrease of the Cat2 signal was observed. Therefore the changes of Cat2 peak height with increasing cell count within the range from 0 to 150 000 were measured at all cell cultures used in our experiments (wM4, wM9, wM12 and wM15). The dependences obtained were linear (**wM4**: y = 0.001x + 0.8073, R^2^ = 0.9829; **wM9**: y = 0.0026x - 0.1156, R^2^ = 0.9999; **wM12**: y = 0.0028x + 0.494 R^2^ = 0.9981; **wM15**: y = 0.0014x + 1.5362, R^2^ = 0.9751), see in Fig. 2B. By dilution of the samples we estimated the lowest count of the cells, we were able to quantify, particularly for wM4: 50 cells, wM9: 7 cells, wM12: 5 cells, and for wM15: 23 cells in 100 μl. On the account of the fact that all samples analysed were diluted 1 000 times as optimized above, the detection limit of the cell count determined can be estimated as 0.005 - 0.05 of a cell. Moreover the metallothionein content in single melanoma cell lines was determined at cell count of 10 000. The metallothionein content varied within the range from 4.2 to 11.2 μM. The lowest content of MT (4.2 μM) was determined at the cell line wM4, the highest (11.2 μM) at the cell line wM12 (Fig. 2C). The contents of MT determined at the cell lines were significantly higher (p < 0.05) compared to control cells without symptoms of tumour transformation, in which MT levels were very low (1.5 ± 0.1 μM).

### 3.3 Metallothionein content in minipig melanoma tissues

At the Institute of Animal Physiology and Genetics in Libechov, Czech Republic, the MeLiM (***Me***lanoma-bearing ***Li***bechov ***M***inipig) strain with hereditary melanoma has been established [82,83]. A high tumour incidence (in 57% of animals), typical progressive growth (about 34% animals die usually within the first 2 months of age), development of metastases in distant organs, and the existence of various multiple pigment lesions (naevi, superficial spreading melanoma and nodular melanoma) make the MeLiM strain a good model of human disease [82,84-89].

**Figure 3.**
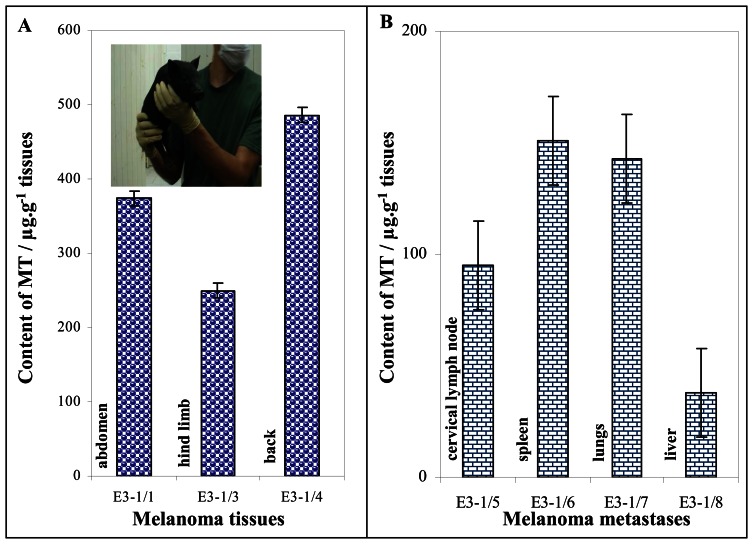
MT content in malignant melanoma localized on abdomen, back limb and dorsum of the MeLiM minipig; in inset: photography of MeLiM piglet (**A**). MT content in metastases from cervical lymph node, spleen, lungs and liver (**B**).

Melanoma tissues from one highly affected MeLiM piglet taken shortly before the death due to tumour progression (age 1 month) were used in this study (inset in Fig. 3). Three primary skin nodular melanomas and samples from the cervical lymph node and visceral organs with metastases (liver, lungs, and spleen) were analysed. Using AdTS DPV Brdicka reaction it was possible to determine the MT content in the sampled tissues. We found out that the average MT content in the tissues samples from malignant melanomas localized on abdomen, back limb and dorsum was of 370 ± 80 μg·g^-1^ of the tissue (Fig. 3A). Each measurement was performed in triplicates; the relative standard deviation was not higher than 10 %. The melanoma metastases were present in the most of organs such as the liver, spleen and lymph nodes. The samples of metastases (weight from 50 to 100 mg) were prepared by preparation needle and processed according the procedure mentioned in “Materials and Methods” section. The MT levels determined in metastases in spleen, lungs and liver were within the range from 40 to 160 μg·g^-1^ of the tissue, the average level was 110 ± 40 μg·g^-1^ of the tissue (Fig. 3B). The MT level in metastases was 3-times lower than in primary tumours. This phenomenon can be associated with poor resolution of metastatic tissue, because the size of metastases was a few millimetres only. Nevertheless MT level at both melanomas and their metastases were significantly higher (p < 0.05) compared to MT level in a healthy tissue. The MT level in healthy tissues is not higher than 10-20 μg·g^-1^ of the tissue.

### 3.4 Determination of metallothionein from the patients with melanoma

Based on the previously published data MT could be considered as a potential prognostic and diagnostic marker of tumour diseases [2,8,13]. Thus metallothionein gene has been intensively studying. We can recognize the DNA sequence where we can find a promoter region called as MRE (Metal responsible element) and MT coding sequence. The MT genes are consisted from several exons (e.g. MT2 gene is consisted from five exons). MT, which is translated from a MT gene, controls homeostasis of metal ions, thereby, could influence an expression of number of other genes (e.g TP53), which could lead to a cell proliferation and to a cancer [90]. In addition the DNA region called MRE could be influenced not only by metal ions but also by biological active proteins. It could be assume also that transcription and/or translation of MT could be changed by different expression of other genes (an influence on responsible elements of GRE and/or ARE). On the other hand the influence of number of proteins, heavy metals and others compounds and processes on the elements have not been proved.

Skin melanoma is the most frequently occurring type of the melanoma. The mucous membrane melanoma is very rare. In spite of relatively low incidence of the uveal melanoma, it is the most occurred tumour disease of eye. The incidence of melanoma increases assiduously both at men and women. It occurs practically throughout the whole productive age with the highest incidence from 30 to 70 years. The high attention has been paying to this malignancy due to its histological origin. The investigations are especially aimed on its relation to different cells of immune system. Recently the studies evaluating the level of metallothionein and the progression of the disease have been published [25,28,30]. The relation between the increased MT level in blood and markedly worse prognosis (the shortening of survival time) has been also studied [27,91].

**Figure 4.**
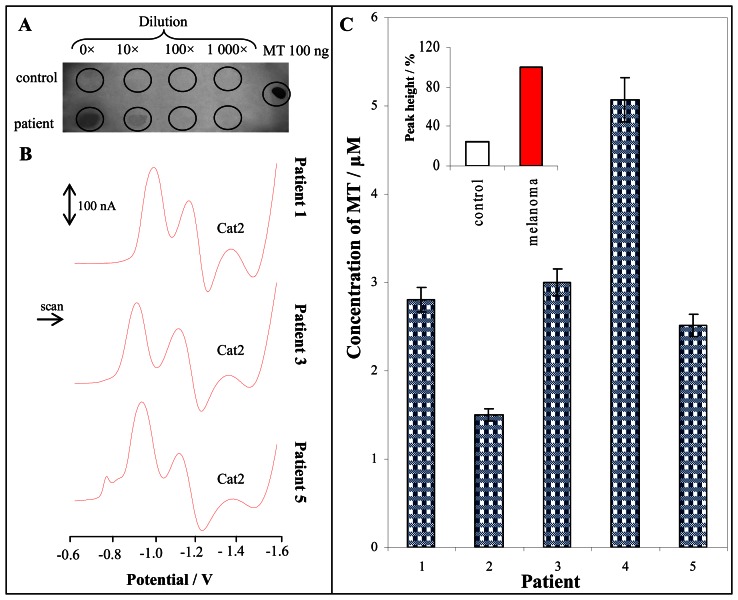
Immunological detection of MT on PVDF membrane, sample volume: 1 μl, visualization: AEC (**A**). Typical DP voltammograms of blood serum from patients with malignant melanoma (patient 1, 3 and 5) (**B**). MT content in patients with malignant melanoma, in inset: MT level at healthy persons (n = 20) and patients with malignant melanoma (n = 5) (**C**). The samples were processed according to procedure mentioned in “Materials and Methods” section. The measurement of 5 μl of 1000 times diluted sample by 0.2 M phosphate buffer (pH 6.8) was performed by AdTS DPV Brdicka reaction.

Here, we determined the MT level in blood serum of patients with skin malignant melanoma. The samples from five patients were obtained. The samples were processed according to procedure mentioned in “Materials and Methods” section. We were interested in the issue if the immunological methods allow us to detect MT in the samples. On PVDF membrane blood samples of the patients with malignant melanoma and healthy volunteers in serial dilutions were applied. The developing of colour reaction was possible to detect only at patients with tumour in concentrated and 10 times diluted sample (Fig. 4A). MT level in healthy volunteers blood serum samples was under detection limit of immunochemical method. Moreover the immunological detection indicates clearly the MT presence in serum of patients with melanoma, but its quantification using this method is rather difficult.

For the purpose to detect MT in blood serum samples of healthy volunteers Brdicka reaction is very advantageous allowing us a quick and relatively precise MT quantification. Based on the abovementioned data the sample was 1 000 times diluted by the phosphate buffer (0.1 M, pH 6.8) and consequently analyzed by AdTS DPV Brdicka reaction. Typical DP voltammograms are shown in Fig. 4B. The signals of MT were well developed in all obtained voltammograms. Each analysis was repeated five times. The relative standard deviation did not exceed 3 %. The average MT level in the patients samples was 3.0 ± 0.8 μM, whereas MT level at 5 patients was higher than 1 μM and at 4 patients the MT level exceeded the 2 μM (Fig. 4C). The MT level determined at cancer patients was higher in comparison to control group (0.8 ± 0.4 μM). The difference between the MT level in blood serum of patients with malignant melanoma and control group is more than 70 % (100 % refers to MT concentration of 3 μM), which is significant at p < 0.05. The mentioned electroanalytical method is a promising analytical tool for the determination of MT level at patients with tumour diseases.

## 4. Conclusion

Great attention on analytical determination of peptides and proteins is paid. Electrochemical methods represent an excellent tool for such studies. As we report in the paper, adsorptive transfer stripping technique coupled with differential pulse voltammetry Brdicka reaction can be used for quantification of metallothioneins in cells, tissues and blood serum samples. The level of these proteins as possible tumour disease markers in melanoma cells and tissues, and blood serum from patients with melanoma was significantly enhanced compared to non-malignant samples. Employing of this electrochemical method in clinical practise seems to be very promising.

## References

[1] Kagi J.H.R., Schaffer A. (1988). Biochemistry of Metallothionein. Biochemistry.

[2] Kizek R., Vacek J., Adam V., Vojtesek B. (2004). Metallothionein - Cisplatin and anticancer therapy. Klin. Biochem. Metab..

[3] Studnickova M., Turanek J., Zabrsova H., Krejci M., Kysel M. (1997). Rat liver metallothioneins are metal dithiolene clusters. J. Electroanal. Chem..

[4] Hamer D.H. (1986). Metallothionein. Annu. Rev. Biochem..

[5] Cousins R.J. (1985). Absorption, Transport, and Hepatic-Metabolism of Copper and Zinc - Special Reference to Metallothionein and Ceruloplasmin. Physiol. Rev..

[6] Kagi J.H.R. (1991). Overview of Metallothionein. Method Enzymol..

[7] Roesijadi G. (1992). Metallothioneins in Metal Regulation and Toxicity in Aquatic Animals. Aquat. Toxicol.

[8] Theocharis S.E., Margeli A.P., Klijanienko J.T., Kouraklis G.P. (2004). Metallothionein expression in human neoplasia. Histopathology.

[9] Dutsch-Wicherek M., Popiela T.J., Klimek M., Rudnicka-Sosin L., Wicherek L., Oudinet J.P., Skladzien J., Tomaszewska R. (2005). Metallothionein stroma reaction in tumor adjacent healthy tissue in head and neck squamous cell carcinoma and breast adenocarcinoma. Neuroendocrinol. Lett..

[10] Klimek M., Wicherek L., Galazka K., Tetlak T., Popiela T.J., Kulczycka M., Rudnicka-Sosin L., Dutsch-Wicherek M. (2005). Cycle dependent expression of endometrial metallothionein. Neuroendocrinol. Lett..

[11] Klimek R. (2001). Biology of cancer: Thermodynamic answers to some questions. Neuroendocrinol. Lett..

[12] Kukacka J., Vajtr D., Huska D., Prusa R., Houstava L., Samal F., Diopan V., Kotaska K., Kizek R. (2006). Blood Metallothionein, Neuron Specific Enolase, And Protein S100B In Patients With Trauma Brain Injury. Neuroendocrinol. Lett..

[13] Prusa R., Blastik O., Potesil D., Trnkova L., Zehnalek J., Adam V., Petrlova J., Jelen F., Kizek R. (2005). Analytic method for determination of metallothioneins as tumor markers. Clin. Chem..

[14] Prusa R., Kizek R., Vacek J., Trnkova L., Zehnalek J. (2004). Study of relationship betwenn metallothionein and heavy metals by CPSA method. Clin. Chem..

[15] Prusa R., Petrlova J., Kukacka J., Adam V., Sures B., Beklova M., Kizek R. (2006). Study of interaction of glutathiones and metallothionein with cytostatics. Clin. Chem..

[16] Prusa R., Svoboda M., Blastik O., Adam V., Zitka O., Beklova M., Eckschlager T., Kizek R. (2006). Increase in content of metallothionein as marker of resistence to cisplatin treatment. Clin. Chem..

[17] Dziegiel P., Suder E., Surowiak P., Kornafel J., Zabel M. (2002). Expression of metallothionein in synovial sarcoma cells. Appl. Immunohistochem..

[18] Goldmann T., Moorkamp A., Wiedorn K.H., Suter L., Otto F. (2001). The prognostic value of the expression of collagenase IV, cathepsin D and metallothionein in squamous cell carcinomas of the skin determined by immunohistochemistry. Arch. Dermatol. Res..

[19] Goldmann T., Ribbert D., Suter L., Brode M., Otto F. (1998). Tumor characteristics involved in the metastatic behaviour as an improvement in primary cutaneous melanoma prognostics. J. Exp. Clin. Cancer Res..

[20] Jin R.X., Huang J.X., Tan P.H., Bay B.H. (2004). Clinicopathological significance of metallothioneins in breast cancer. Pathol. Oncol. Res..

[21] Mitropoulos D., Kyroudi-Voulgari A., Theocharis S., Serafetinides E., Moraitis E., Zervas A., Kittas C. (2005). Prognostic significance of metallothionein expression in renal cell carcinoma. World J. Gastroenterol..

[22] Ohshio G., Imamura T., Okada N., Wang Z.H., Yamaki K., Kyogoku T., Suwa H., Yamabe H., Imamura M. (1996). Immunohistochemical study of metallothionein in pancreatic carcinomas. J. Cancer Res. Clin. Oncol..

[23] Sauerbrey A., Zintl F., Volm M. (1994). Expression of Metallothionein in Initial and Relapsed Childhood Acute Lymphoblastic-Leukemia. Ann. Hematol..

[24] Shukla V.K., Aryya N.C., Pitale A., Pandey M., Dixit V.K., Reddy C.D., Gautam A. (1998). Metallothionein expression in carcinoma of the gallbladder. Histopathology.

[25] Sugita K., Yamamoto O., Asahi M. (2001). Immunohistochemical analysis of metallothionein expression in malignant melanoma in Japanese patients. Am. J. Dermatopathol..

[26] Surowiak P., Materna V., Kaplenko I., Spaczynski M., Dietel M., Lage H., Zabel M. (2005). Augmented expression of metallothionein and glutathione S-transferase pi as unfavourable prognostic factors in cisplatin-treated ovarian cancer patients. Virchows Arch..

[27] Zelger B., Hittmair A., Schir M., Ofner C., Ofner D., Fritsch P.O., Bocker W., Jasani B., Schmid K.W. (1993). Immunohistochemically Demonstrated Metallothionein Expression in Malignant-Melanoma. Histopathology.

[28] Weinlich G., Eisendle K., Hassler E., Baltaci M., Fritsch P.O., Zelger B. (2006). Metallothionein - overexpression as a highly significant prognostic factor in melanoma: a prospective study on 1270 patients. Br. J. Cancer.

[29] Meyskens F.L., Farmer P.J., Anton-Culver H. (2004). Etiologic pathogenesis of melanoma: A unifying hypothesis for the missing attributable risk. Clin. Cancer Res..

[30] Weinlich G., Bitterlich W., Mayr V., Fritsch P.O., Zelger B. (2003). Metallothionein-overexpression as a prognostic factor for progression and survival in melanoma. A prospective study on 520 patients. Br. J. Dermatol..

[31] Sarangarajan R., Apte S.P. (2006). The polymerization of melanin: a poorly understood phenomenon with egregious biological implications. Melanoma Res..

[32] Happle R., Traupe H., Vakilzadeh F., Macher E. (1982). Arguments in Favor of a Polygenic Inheritance of Precursor Nevi. J. Am. Acad. Dermatol..

[33] Kamb A., Shattuckeidens D., Eeles R., Liu Q., Gruis N.A., Ding W., Hussey C., Tran T., Miki Y., Weaverfeldhaus J., McClure M., Aitken J.F., Anderson D.E., Bergman W., Frants R., Goldgar D.E., Green A., Maclennan R., Martin N.G., Meyer L.J., Youl P., Zone J.J., Skolnick M.H., Cannonalbright L.A. (1994). Analysis of the P16 Gene (Cdkn2) as a Candidate for the Chromosome 9p Melanoma Susceptibility Locus. Nature Genet..

[34] Gruis N.A., Vandervelden P.A., Sandkuijl L.A., Prins D.E., Weaverfeldhaus J., Kamb A., Bergman W., Frants R.R. (1995). Homozygotes for Cdkn2 (P16) Germline Mutation in Dutch Familial Melanoma Kindreds. Nature Genet..

[35] Vasen H.F.A., Gruis N.A., Frants R.R., van der Velden P.A., Hille E.T.M., Bergman W. (2000). Risk of developing pancreatic cancer in families with familial atypical multiple mole melanoma associated with a specific 19 deletion of p16 (p16-Leiden). Int. J. Cancer.

[36] Kefford R., Bishop J.N., Tucker M., Bressac-de-Paillerets B., Bianchi-Scarra G., Bergman W., Goldstein A., Puig S., Mackie R., Elder D., Hansson J., Hayward N., Hogg D., Olsson H. (2002). Genetic testing for melanoma. Lancet Oncol..

[37] Goldstein A.M., Chan M., Harland M., Hayward N.K., Demenais F., Bishop D.T., Azizi E., Bergman W., Bianchi-Scarra G., Bruno W., Calista D., Albright L.A.C., Chaudru V., Chompret A., Cuellar F., Elder D.E., Ghiorzo P., Gillanders E.M., Gruis N.A., Hansson J., Hogg D., Holland E.A., Kanetsky P.A., Kefford R.F., Landi M.T., Lang J., Leachman S.A., MacKie R.M., Magnusson V., Mann G.J., Bishop J.N., Palmer J.M., Puig S., Puig-Butille J.A., Stark M., Tsao H., Tucker M.A., Whitaker L., Yakobson E. (2007). Features associated with germline CDKN2A mutations: a GenoMEL study of melanoma-prone families from three continents. J. Med. Genet..

[38] Alvarado N.E., Cancio I., Hylland K., Marigomez I., Soto M. (2007). Immunolocalization of metallothioneins in different tissues of turbot (Scophthalmus maximus) exposed to Cd. Histol. Histopath..

[39] Alves S., Cardoso S.V., Bernardes V.D.F., Machado V.C., Mesquita R.A., do Carmo M.A.V., Aguiar M.C.F. (2007). Metallothionein immunostaining in adenoid cystic carcinomas of the salivary glands. Oral Oncol..

[40] Lobinski R., Chassaigne H., Szpunar J. (1998). Analysis for metallothioneins using coupled techniques. Talanta.

[41] Szpunar J. (2000). Bio-inorganic speciation analysis by hyphenated techniques. Analyst.

[42] Szpunar J., Lobinski R., Prange A. (2003). Hyphenated techniques for elemental speciation in biological systems. Appl. Spectrosc..

[43] Chassaigne H., Lobinski R. (1998). Characterization of metallothionein isoforms by reversed-phase high-performance liquid chromatography with on-line post-column acidification and electrospray mass spectrometric detection. J. Chromatogr. A.

[44] Chassaigne H., Lobinski R. (1998). Characterization of horse kidney metallothionein isoforms by electrospray MS and reversed-phase HPLC-electrospray MS. Analyst.

[45] Chassaigne H., Lobinski R. (1998). Polymorphism and identification of metallothionein isoforms by reversed-phase HPLC with on-line ion spray mass spectrometric detection. Anal. Chem..

[46] Kizek R., Vacek J., Trnkova L., Klejdus B., Havel L. (2004). Application of catalytic reactions on a mercury electrode for metallothionein electrochemical detection. Chem. Listy..

[47] Thompson J.A.J., Cosson R.P. (1984). An improved electrochemical method for the quantification of metallothioneins in marine organisms. Marine Environ. Res..

[48] Dabrio M., Rodriguez A.R., Bordin G., Bebianno M.J., De Ley M., Šestakova I., Vasak M., Nordberg M. (2002). Recent developments in quantification methods for metallothionein. J. Inorg. Biochem..

[49] Sestakova I., Navratil T. (2005). Voltammetric methods in metallothionein research. Bioinorg. Chem. Appl..

[50] Adam V., Blastik O., Krizkova S., Lubal P., Kukacka J., Prusa R., Kizek R. (2008). Application of the Brdicka reaction in determination of metallothionein in patients with tumours. Chem. Listy.

[51] Adam V., Beklova M., Pikula J., Hubalek J., Trnkova L., Kizek R. (2007). Shapes of differential pulse voltammograms and level of metallothionein at different animal species. Sensors.

[52] Kukacka J., Vajtr D., Huska D., Prusa R., Houstava L., Samal F., Diopan V., Kotaska K., Kizek R. (2006). Blood metallothionein, neuron specific enolase, and protein S100B in patients with traumatic brain injury. Neuroendocrinol. Lett..

[53] Adam V., Krizkova S., Zitka O., Trnkova L., Petrlova J., Beklova M., Kizek R. (2007). Determination of apo-metallothionein using adsorptive transfer stripping technique in connection with differential pulse voltammetry. Electroanalysis.

[54] Petrlova J., Potesil D., Mikelova R., Blastik O., Adam V., Trnkova L., Jelen F., Prusa R., Kukacka J., Kizek R. (2006). Attomole voltammetric determination of metallothionein. Electrochim. Acta.

[55] Yang M.L., Zhang Z.J., Hu Z.B., Li J.H. (2006). Differential pulse anodic stripping voltammetry detection of metallothionein at bismuth film electrodes. Talanta.

[56] Adam V., Petrlova J., Potesil D., Zehnalek J., Sures B., Trnkova L., Jelen F., Kizek R. (2005). Study of metallothionein modified electrode surface behavior in the presence of heavy metal ions-biosensor. Electroanalysis.

[57] Lopez M.J., Arino C., Diaz-Cruz S., Diaz-Cruz J.M., Tauler R., Esteban M. (2003). Voltammetry assisted by multivariate analysis as a tool for speciation of metallothioneins: Competitive complexation of alpha- and beta-metallothionein domains with cadmium and zinc. Environ. Sci. Technol..

[58] Diaz-Cruz M.S., Lopez M.J., Diaz-Cruz J.M., Esteban M. (2002). Comparison of the zinc-cadmium exchange properties of the metallothionein related peptide {Lys-Cys-Thr-Cys-Cys-Ala} and a zinc-containing metallothionein: study by voltammetry and multivariate curve resolution. J. Electroanal. Chem..

[59] Sestakova I., Mader P. (2000). Voltammetry on mercury and carbon electrodes as a tool for studies of metallothionein interactions with metal ions. Cell. Mol. Biol..

[60] Erk M., Raspor B. (2000). Advantages and disadvantages of voltammetric method in studying cadmium-metallothionein interactions. Cell. Mol. Biol..

[61] Erk M., Ivankovic D., Raspor B., Pavicic J. (2002). Evaluation of different purification procedures for the electrochemical quantification of mussel metallothioneins. Talanta.

[62] Raspor B. (2001). Elucidation of the mechanism of the Brdicka reaction. J. Electroanal. Chem..

[63] Brdicka R. (1937). Polarographic investigation in serological cancer diagnosis. Nature.

[64] Brdicka R. (1937). Application of the polarographic effect of proteins in cancer diagnosis. Nature.

[65] Huska D., Krizkova S., Beklova M., Havel L., Zehnalek J., Diopan V., Adam V., Zeman L., Babula P., Kizek R. (2008). Influence of cadmium(II) ions and brewery sludge on metallothionein level in earthworms (Eisenia fetida) - Biotransforming of toxic wastes. Sensors.

[66] Kizek R., Trnkova L., Palecek E. (2001). Determination of metallothionein at the femtomole level by constant current stripping chronopotentiometry. Anal. Chem..

[67] Kizek R., Vacek J., Trnkova L., Klejdus B., Havel L. (2004). Application of catalytic reactions on a mercury electrode for electrochemical detection of metallothioneins. Chem. Listy.

[68] Krizkova S., Zitka O., Adam V., Beklova M., Horna A., Svobodova Z., Sures B., Trnkova L., Zeman L., Kizek R. (2007). Possibilities of electrochemical techniques in metallothionein and lead detection in fish tissues. Czech J. Anim. Sci..

[69] Ostatna V., Palecek E. (2008). Native, denatured and reduced BSA - Enhancement of chronopotentiometric peak H by guanidinium chloride. Electrochim. Acta.

[70] Petrlova J., Krizkova S., Zitka O., Hubalek J., Prusa R., Adam V., Wang J., Beklova M., Sures B., Kizek R. (2007). Utilizing a chronopotentiometric sensor technique for metallothionein determination in fish tissues and their host parasites. Sens. Actuator B-Chem..

[71] Serrano N., Sestakova I., Diaz-Cruz J.M. (2006). Constant current stripping chronopotentiometry for the study of adsorbing inert and electrochemically nonreversible metal complexes at low concentrations: Application to Cd and Zn metallothioneins. Electroanalysis.

[72] Serrano N., Sestakova I., Diaz-Cruz J.M., Arino C. (2006). Adsorptive accumulation in constant current stripping chronopotentiometry as an alternative for the electrochemical study of metal complexation by thiol-containing peptides. J. Electroanal. Chem..

[73] Sestakova I., Kopanica M., Havran L., Palecek E. (2000). Constant current chronopotentiometric stripping analysis of Cd-metallothionein on carbon and mercury electrodes. Comparison with voltammetry. Electroanalysis.

[74] Strouhal M., Kizek R., Vecek J., Trnkova L., Nemec M. (2003). Electrochemical study of heavy metals and metallothionein in yeast Yarrowia lipolytica. Bioelectrochemistry.

[75] Tomschik M., Havran L., Fojta M., Palecek E. (1998). Constant current chronopotentiometric stripping analysis of bioactive peptides at mercury and carbon electrodes. Electroanalysis.

[76] Tomschik M., Havran L., Palecek E., Heyrovsky M. (2000). The “presodium” catalysis of electroreduction of hydrogen ions on mercury electrodes by metallothionein. An investigation by constant current derivative stripping chronopotentiometry. Electroanalysis.

[77] Trnkova L., Kizek R., Vacek J. (2002). Catalytic signal of rabbit liver metallothionein on a mercury electrode: a combination of derivative chronopotentiometry with adsorptive transfer stripping. Bioelectrochemistry.

[78] Lauerova L., Dusek L., Simickova M., Kocak I., Vagundova M., Zaloudik J., Kovarik J. (2002). Malignant melanoma associates with Th1/Th2 imbalance that coincides with disease progression and immunotherapy response. Neoplasma.

[79] Supalkova V., Petrek J., Baloun J., Adam V., Bartusek K., Trnkova L., Beklova M., Diopan V., Havel L., Kizek R. (2007). Multi-instrumental investigation of affecting of early somatic embryos of Spruce by cadmium(II) and lead(II) ions. Sensors.

[80] Erk M., Ivanković D., Raspor B., Pavičić J. (2002). Evaluation of different purification procedures for the electrochemical quantification of mussel metallothioneins. Talanta.

[81] Petrlova J., Potesil D., Zehnalek J., Sures B., Adam V., Trnkova L., Kizek R. (2006). Cisplatin electrochemical biosensor. Electrochim. Acta.

[82] Fortyn K., Hruban V., Horak V., Tichy J. (1998). Exceptional occurrence and extent of malignant melanoma in pig. Vet. Med..

[83] Horak V., Fortyn K., Hruban V., Klaudy J. (1999). Hereditary melanoblastoma in miniature pigs and its successful therapy by devitalization technique. Cell. Mol. Biol..

[84] Borovansky J., Horak V., Elleder M., Fortyn K., Smit N.P.M., Kolb A.M. (2003). Biochemical characterization of a new melanoma model the minipig MeLiM strain. Melanoma Res..

[85] Geffrotin C., Crechet F., Le Roy P., Le Chalony C., Leplat J.J., Iannuccelli N., Barbosa A., Renard C., Gruand J., Milan D., Horak V., Tricaud Y., Bouet S., Franck M., Frelat G., Vincent-Naulleau S. (2004). Identification of five chromosomal regions involved in predisposition to melanoma by genome-wide scan in the MeLiM swine model. Int. J. Cancer.

[86] Vincent-Naulleau S., Le Chalony C., Leplat J.J., Bouet S., Bailly C., Spatz A., Vielh P., Avril M.F., Tricaud Y., Gruand J., Horak V., Frelat G., Geffrotin C. (2004). Clinical and histopathological characterization of cutaneous melanomas in the melanoblastoma-bearing Libechov minipig model. Pigm. Cell. Res..

[87] Svoboda M., Eichlerova K., Horak V., Hradecky J. (2005). Development of haematological indices in melanoma-bearing Libechov Minipigs. Acta Vet. BRNO.

[88] Le Chalony C., Renard C., Vincent-Naulleau S., Crechet F., Leplat J.J., Tricaud Y., Horak V., Gruand J., Le Roy P., Frelat G., Geffrotin C. (2003). CDKN2A region polymorphism and genetic susceptibility to melanoma in the melim swine model of familial melanoma. Int. J. Cancer.

[89] Geffrotin C., Horak V., Crechet F., Tricaud Y., Lethias C., Vincent-Naulleau S., Vielh P. (2000). Opposite regulation of tenascin-C and tenascin-X in MeLiM swine heritable cutaneous malignant melanoma. Biochim. Biophys. Acta-Gen. Subj..

[90] Potesil D., Mikelova R., Adam V., Kizek R., Prusa R. (2006). Change of the protein p53 electrochemical signal according to its structural form - Quick and sensitive distinguishing of native, denatured, and aggregated form of the “guardian of the genome”. Protein J..

[91] Breazeale R.I., Fishburn J., Buchanan T., Stone J. (1996). Overexpression of metallothionein and survival in uveal melanoma. Invest. Ophthalmol. Vis. Sci..

